# Supplementary material for: Seasonal Variability in the Prevalence of DWV Strains in Individual Colonies of European Honeybees in Hawaii
Source: Insects. 2024 Mar 23;15(4):219. doi: 10.3390/insects15040219 (PMC11050578; doi:10.3390/insects15040219)
Supplement: Supplementary file 1 [file insects-15-00219-s001.zip › insects-2924984-supplementary.pdf]

Table S1. DWV A/B genome equivalent of each colony throughout the study period.

Year	Month	Hive	Genome equivalent	
			DWV-A	DWV-B
2018	Jan	1	1.89E+8	1.61E+7
2018	May	1	5.74E+8	5.57E+4
2018	Sep	1	1.30E+8	1.66E+7
2018	Jan	2	NA	NA
2018	May	2	2.67E+8	6.75E+6
2018	Sep	2	8.49E+7	NA
2018	Jan	3	6.70E+7	7.88E+5
2018	May	3	2.05E+8	3.58E+4
2018	Sep	3	1.42E+8	NA
2018	Jan	4	9.59E+7	1.12E+8
2018	May	4	1.98E+8	NA
2018	Sep	4	4.12E+7	NA
2018	Jan	5	2.36E+7	NA
2018	May	5	3.46E+5	NA
2018	Sep	5	1.76E+8	NA
2018	Jan	6	1.92E+8	NA
2018	May	6	3.87E+8	1.08E+7
2018	Sep	6	5.63E+8	6.12E+6
2018	Jan	7	9.00E+7	NA
2018	May	7	1.30E+9	1.87E+5
2018	Sep	7	5.55E+8	2.56E+8
2019	Jan	7	5.43E+8	3.45E+5
2019	May	7	1.26E+6	4.24E+5
2018	Jan	8	3.28E+8	NA
2018	May	8	1.97E+6	2.05E+3
2018	Sep	8	9.27E+7	9.51E+7
2019	Jan	8	3.47E+9	8.20E+8
2019	May	8	2.83E+5	NA
2019	Sep	8	3.08E+8	NA
2018	Jan	9	NA	NA
2018	May	9	2.63E+8	1.44E+7
2018	Sep	9	1.54E+7	NA
2019	Jan	9	3.50E+8	NA
2019	May	9	2.58E+5	NA
2019	Sep	9	1.32E+6	1.98E+6
2018	Jan	10	1.28E+4	NA
2018	May	10	8.78E+7	NA
2018	Sep	10	1.56E+7	NA
2019	Jan	10	4.80E+8	NA

2019	May	10	5.15E+7	1.14E+6
2019	Sep	10	3.75E+8	1.83E+6
2019	Jan	11	1.63E+8	1.40E+6
2019	May	11	3.52E+7	2.70E+5
2019	Sep	11	6.02E+8	NA
2019	Jan	12	7.14E+8	1.48E+5
2019	May	12	1.19E+7	1.24E+5
2019	Sep	12	6.39E+8	NA
2019	Jan	13	2.86E+7	NA
2019	May	13	4.09E+6	1.80E+5
2019	Sep	13	3.52E+8	NA
2019	Jan	14	1.34E+8	NA
2019	May	14	NA	6.80E+5
2019	Sep	14	1.37E+5	NA
2019	Jan	15	9.73E+8	7.94E+8
2019	May	15	6.02E+6	3.26E+5
2019	Jan	16	1.37E+8	NA
2019	May	16	NA	1.37E+6
